# A Rounded Cardiac Mass

**DOI:** 10.1016/j.jaccas.2026.109668

**Published:** 2026-08-18

**Authors:** Mazen M. Kawji, Aristides De La Hera, Ying-Chun Lo, Jeffrey Schwartz

**Affiliations:** a Cardiovascular Institute, OSF, Peoria, Illinois, USA; b Saint Elizabeth Medical Center, Ottawa, Illinois, USA; c Department of Laboratory Medicine and Pathology, Mayo Clinic, Rochester, Minnesota, USA; d Department of Thoracic and Cardiovascular Surgery, Loyola University Medical Center, Maywood, Illinois, USA

**Keywords:** echocardiography, electrocardiogram, imaging, shortness of breath

## Abstract

**Background:**

Cardiac lipomas are rare and frequently asymptomatic. However, they are increasingly diagnosed due to advanced imaging modalities.

**Case Summary:**

Here we present a case of a large right atrial interdigitating lipoma in a female teenager. After having vague symptoms for 3 years, she was referred to a cardiologist. An electrocardiogram showed an isolated P pulmonale suggestive of right atrial enlargement. A 2-dimensional echocardiogram showed a large semirounded right atrial mass. A magnetic resonance imaging scan was consistent with a lipoma. Open heart surgery was performed, and the tumor was resected. Pathology confirmed the presence of an interdigitating benign lipoma, an exceedingly rare tumor.

**Discussion:**

About 300 to 400 cases of cardiac lipoma are reported in the literature. Most cardiac lipomas are composed of mature white adipose tissue. They occur most frequently in the right atrium. Although they may be asymptomatic, they may have a wide range of symptoms including sudden death. Surgical resection is sometimes a challenge owing to tumor size and location.

**Take-Home Message:**

Simple diagnostic tools followed by advanced imaging are leading to detecting more and more rare cardiac diseases, including masses. Early intervention could lead to better outcomes.

## Graphical Abstract

**Figure:**
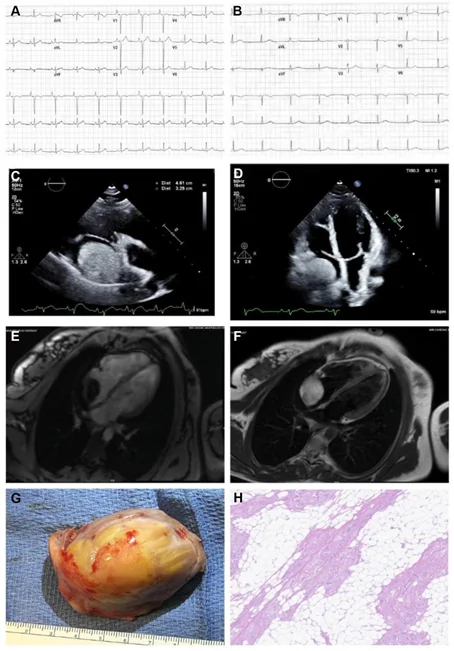

## History of Presentation

A 19-year-old woman was referred to the section of cardiology for evaluation of suspected postural orthostatic tachycardia syndrome. For 3 years, she reported spells of dizziness and lightheadedness after prolonged standing and after long walks. These episodes were associated with heart racing, sweating, shakiness, and occasionally with near syncope, but she never passed out. She described discoloration of her skin with some of these episodes. She denied chest pain but recently experienced shortness of breath, sometimes at rest. She was thought to have anxiety and depression. She did not respond to fluoxetine. Her medical history was otherwise unremarkable.

The physical exam showed a petite, healthy-looking woman. Blood pressure was 94/64 with a regular pulse at 84 and a respiratory rate of 16. No orthostatic changes were noted. Jugular venous pressure was normal. There was no distention of neck veins. Cardiac exam showed no ventricular heaves. There were no murmurs and no extra sounds. The rest of the exam was unremarkable.

## Investigations

An electrocardiogram (ECG) was done at the cardiologist’s office (Figure 1). It showed a normal sinus rhythm with a tall (4 mm equal to 0.4 mV) peaked P-wave in V1 diagnostic of a P pulmonale. The P-wave is less prominent in Lead II and other limb leads. The P-wave axis was normal. The unusual aspect of the ECG is the absence of any other abnormality that may indicate right heart disease. There was no right axis deviation or right bundle branch block. There were no criteria for right ventricular hypertrophy or strain. QRS complexes and T-waves were normal. Criteria of a large right atrium were clearly noted. Complete blood count and chemistry panel were normal. A 2-week event recorder showed no pauses and no atrioventricular block. Intermittent ectopic atrial rhythm was noted, but there were no runs of tachycardia. The patient reported complaints frequently during the study, correlating mostly with normal sinus rhythm. A chest X-ray was normal.

**Figure 1:**
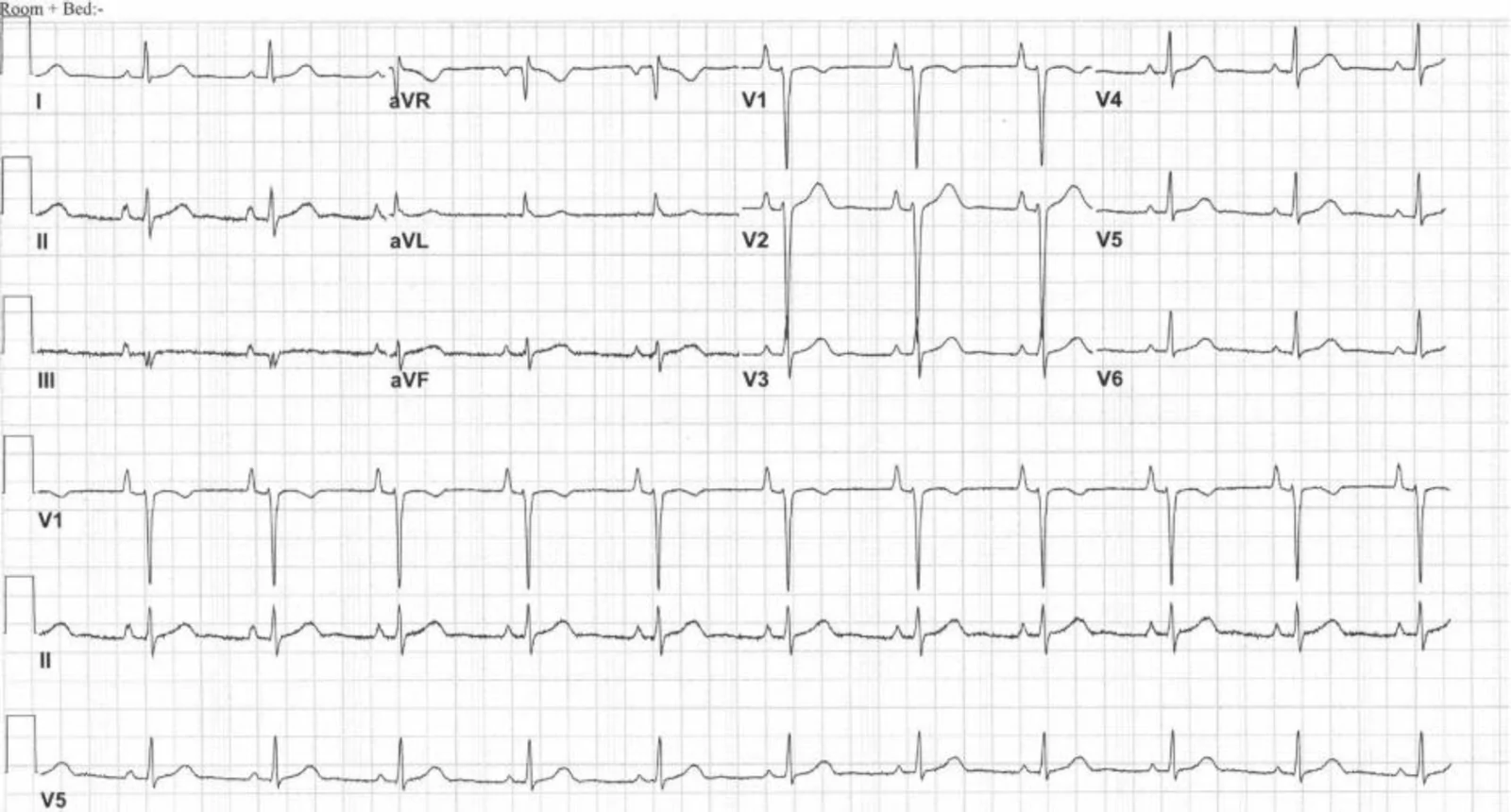
Presenting ECG An electrocardiogram done at the office showed normal sinus rhythm with a peculiar P pulmonale measuring 4 mm in height (0.4 milli volt) in V1, in the absence of right-axis deviation and right ventricular hypertrophy.

**Visual Summary:**
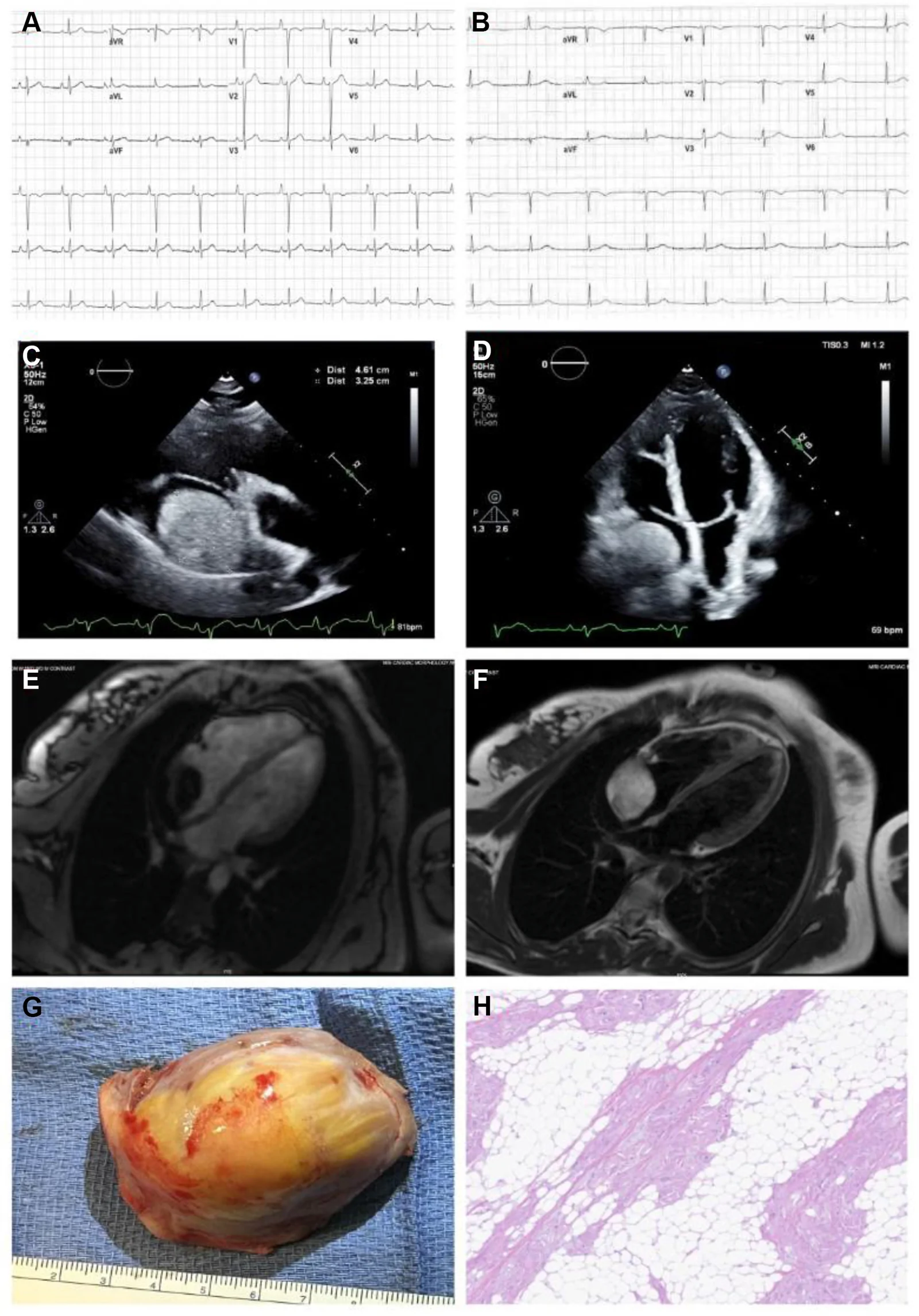
. (A) Presenting electrocardiogram. (B) Postoperative ECG showing resolution of P pulmonale. (C and D) A right atrial mass by echocardiogram. (E and F) Right atrial mass characteristics by cardiac magnetic resonance imaging. (G) Resected atrial mass. (H) Histopathology of the mass.

A transthoracic echocardiogram revealed the diagnosis. It showed a large, echogenic, mobile semi-rounded mass in the right atrium, measuring 4.7 cm by 3.7 cm (Figures 2A and 2B). No apparent stalk was noted. The mass appeared contiguous with the right atrial wall, unrelated to the interatrial septum and the tricuspid valve.

**Figure 2:**
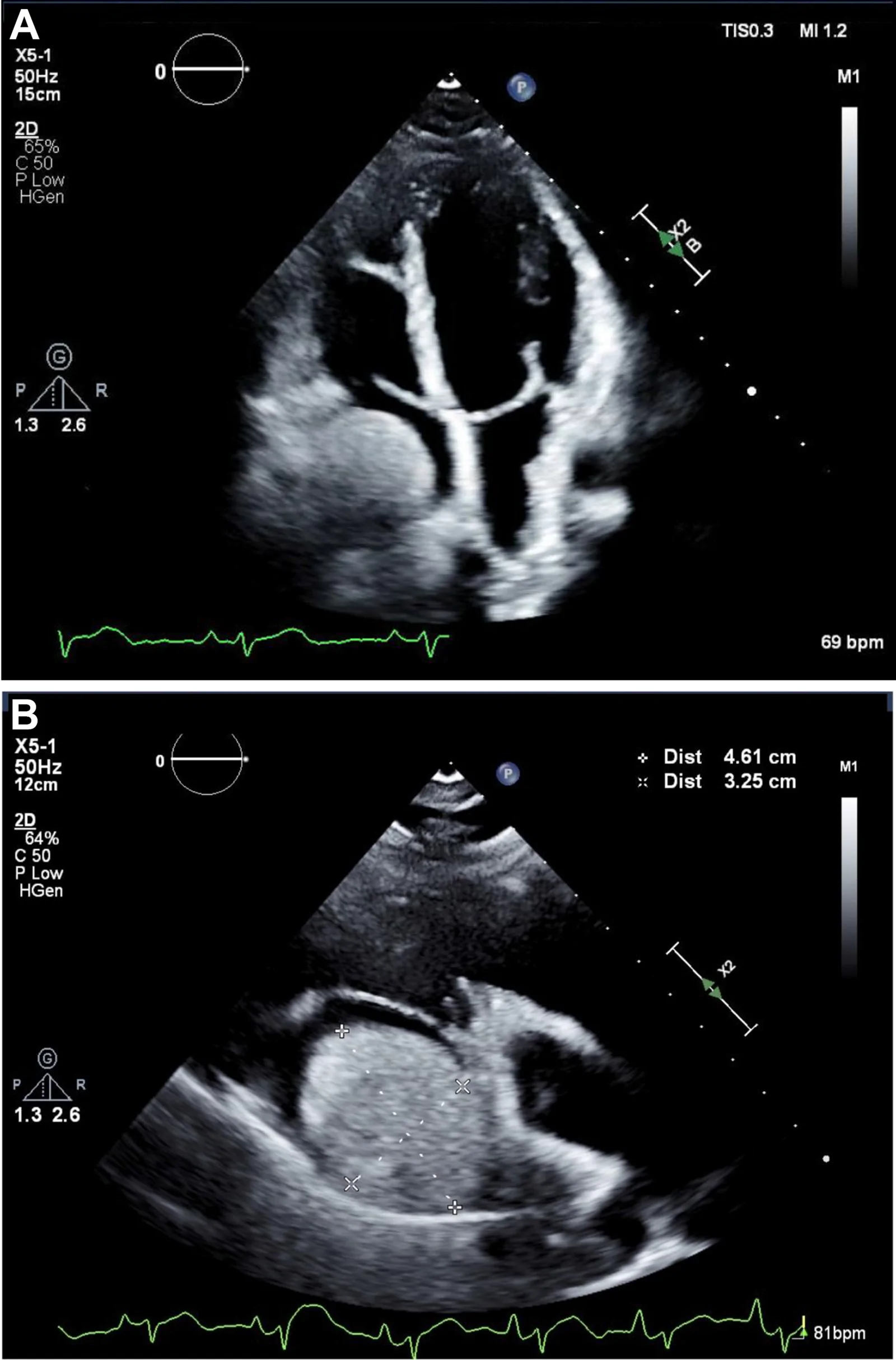
2-D Echocardiogram (A) 4-chamber apical view of the 2-dimensional echocardiogram showed a large, semirounded echogenic mass in the right atrium, measuring 4.7 by 3.3 cm. The mass was not attached to the interatrial septum or the tricuspid valve. (B) A parasternal, short-axis view of the 2-dimensional echocardiogram again showing a large right atrial mass.

A cardiothoracic surgery consultation was promptly arranged. A cardiac magnetic resonance imaging (MRI) showed a broad-based large mass in the right atrium in close contiguity to the posterior wall, which suppressed with fat suppression pulses, characteristic of a lipoma. There was no late gadolinium enhancement. The mass did not appear to invade past the wall of the atrium. It measured 2.5 × 4.6 × 4.0 cm (Figures 3A and 3B).

**Figure 3:**
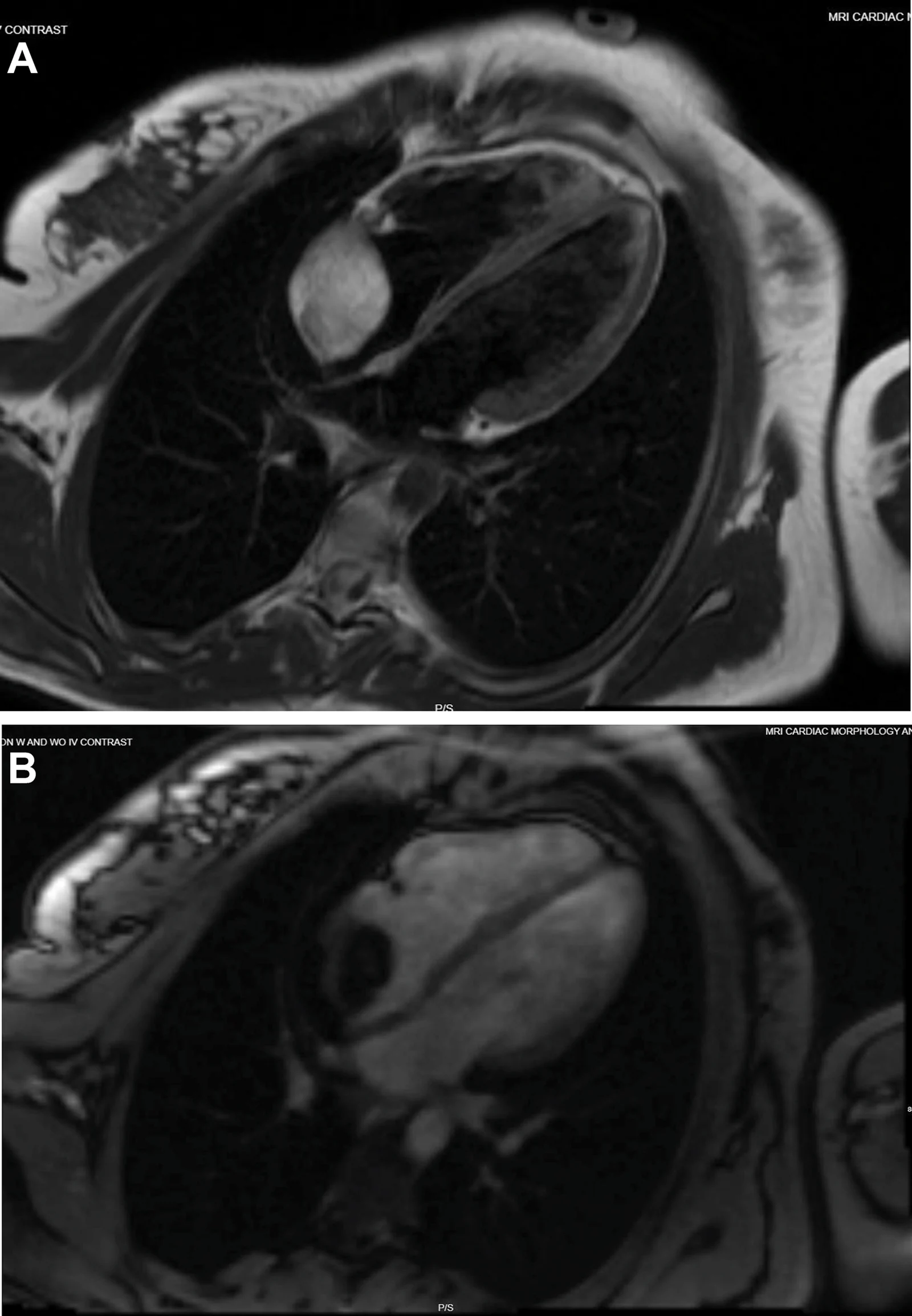
Cardiac MRI Findings (A) Cardiac magnetic resonance imaging with T1 imaging showed a broad base, hyperintense mass in the right atrium. The mass was not attached to the interatrial septum. (B) Cardiac magnetic resonance imaging with fat suppression imaging showed a hypointense mass in the right consistent with a lipoma.

## Differential Diagnosis

The mass lacked a stalk, making myxoma less likely. Metastatic malignancy was a remote possibility; however, the patient did not have any other suspicious symptoms or signs of cancer no change in weight and had normal labs. The mass was not extending into the inferior vena cava, and given the patient’s age, renal cell carcinoma was essentially ruled out. Other cardiac tumors, such as rhabdomyomas and fibroelastomas, were not in the differential diagnosis because they originate within the myocardium or on the valves, respectively. The MRI findings were consistent with a lipoma. The size of the tumor, and the presence of symptoms, led to prompt surgery, without pursuing other imaging modalities.

## Management and Outcome

The mass was resected through a right mini thoracotomy with bovine pericardial reconstruction of the right atrial wall and superior vena conjunction. The yellowish-orange mass was semirounded, measuring almost 5 cm in its longest diameter (Figure 4). Pathology examination showed a benign lipomatous lesion with mature and uniform adipocytes infiltrating and separating myocyte fibers, creating a unique striated appearance (Figures 5A and 5B). The main pathological differential diagnoses were lipomatous hypertrophy of the atrial septum, benign interdigitating lipoma, and liposarcoma. There were no morphological or immunohistochemical features of liposarcoma. The presence of very limited brown fat cells and the patient’s young age made the diagnosis of lipomatous hypertrophy of the interatrial septum much less likely. In addition, imaging studies showed a thin interatrial septum, clearly separated from the tumor. The final diagnosis was a benign interdigitating lipoma, which is exceptionally rare.

**Figure 4:**
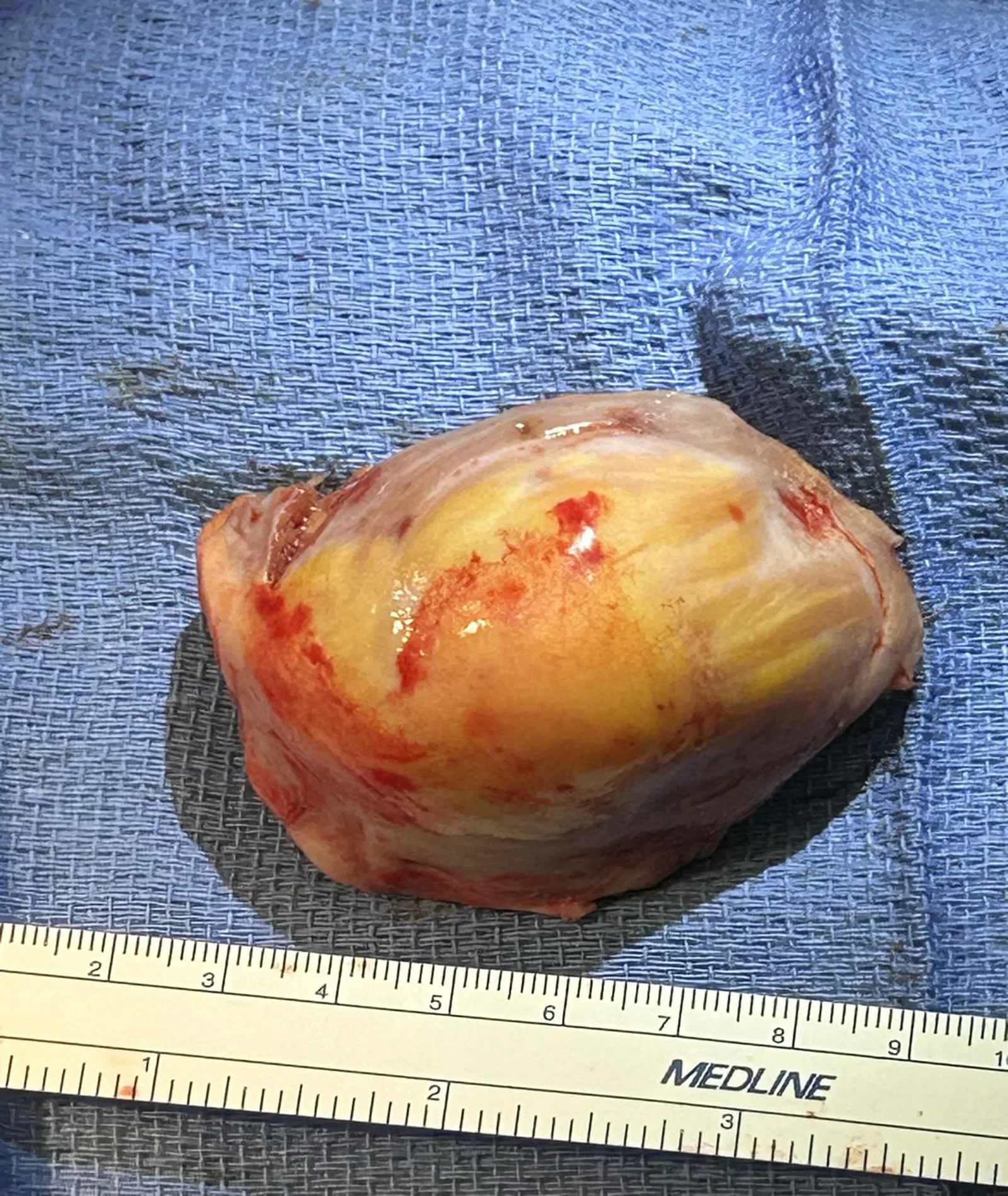
Resected Right Atrial Mass A 4 by 5 cm, yellowish oval mass removed during open heart surgery.

**Figure 5:**
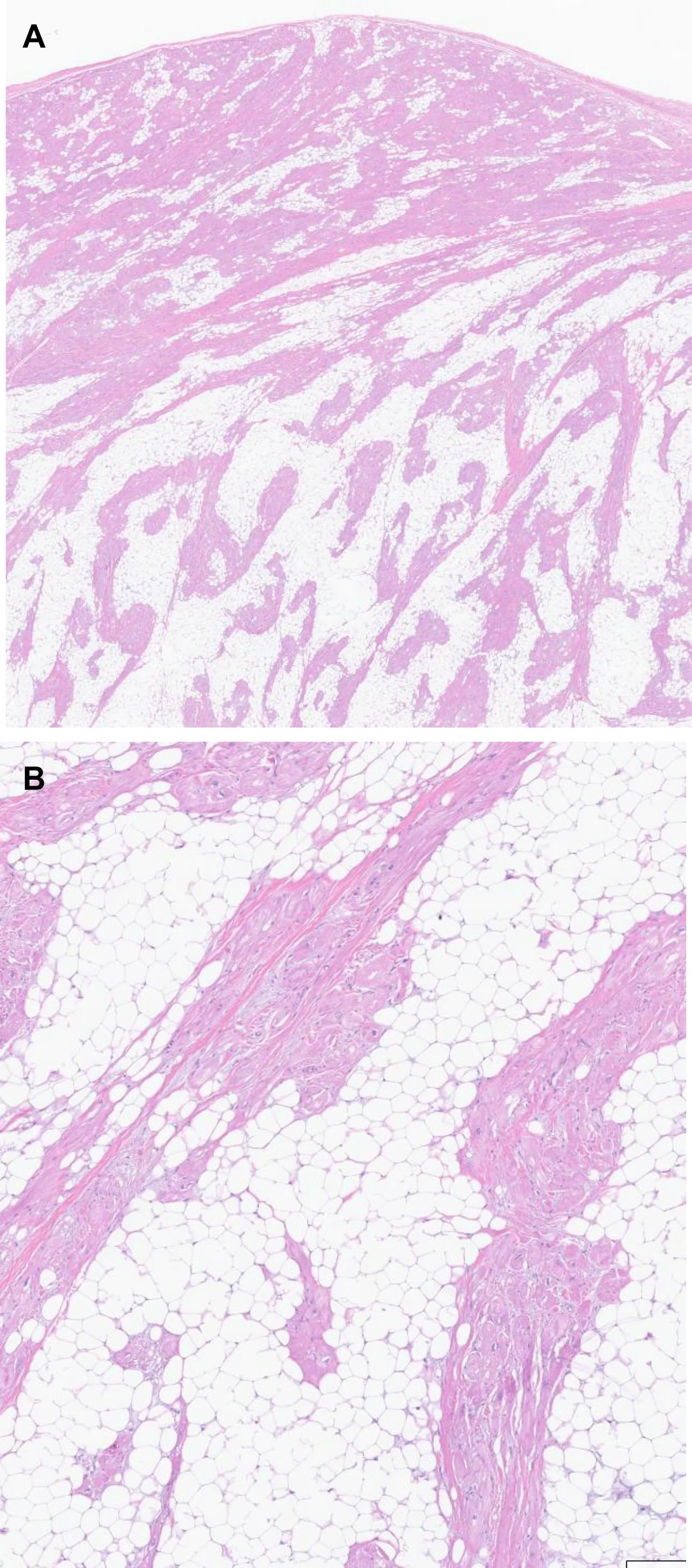
Histopathology Interpretation (A and B) histopathology of the right atrial mass shows mature fat tissue that infiltrates or “interdigitates” between existing heart muscle fibers (myocytes). See text.

Postoperatively, the patient was hemodynamically stable but required narcotics for pain control. She developed transient bradycardia. An ECG showed an ectopic atrial rhythm with small P-waves (Figure 6). The patient was discharged home in stable condition.

**Figure 6:**
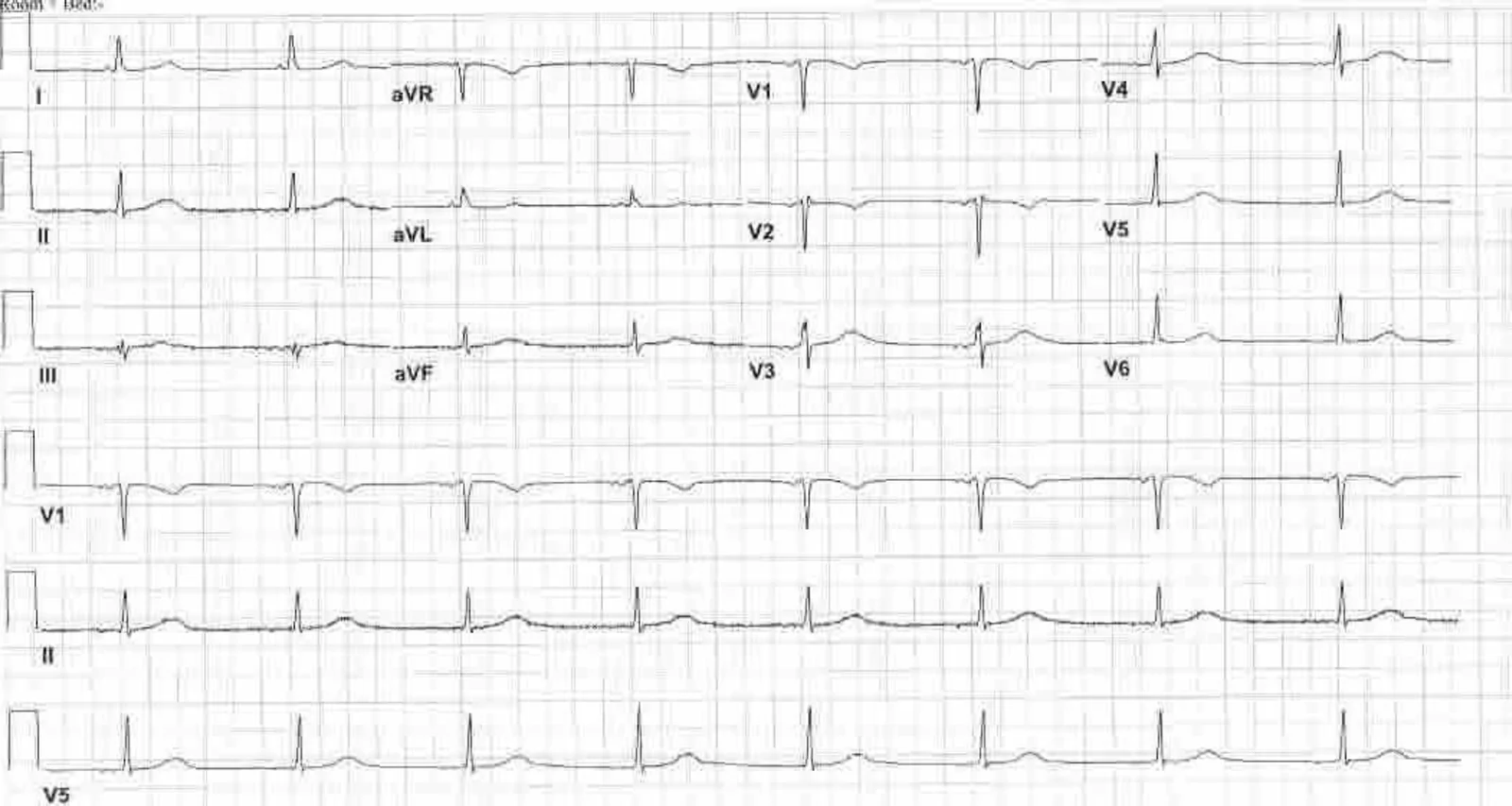
An Electrocardiogram Done Postoperatively Showed Resolution of P Pulmonale The rhythm was more likely ectopic atrial rather than sinus.

A few weeks later, the patient presented with dizziness and near syncope. A repeat event recorder showed frequent sinus pauses up to 5.6 seconds and episodes of severe bradycardia, with the heart rate going down to 28. A dual-chamber pacemaker was implanted.

## Discussion

Primary cardiac tumors are very rare with a prevalence around 0.2%, of which 75% are benign, with myxoma being the most common benign tumor. Other tumors include rhabdomyomas, fibroelastomas, and lipomas. Lipomas represent only 8.4% of primary cardiac tumors.^1^ Malignant liposarcomas are less frequent. However, benign lipomas may infiltrate adjacent cardiac walls, contributing to the difficulty of resecting the entire tumor mass. Cardiac lipoma is mostly circumscribed and epicardial and seen less commonly in the myocardium.

Lipomas occur at all ages and in both genders. Our patient is one of the youngest afflicted reported in the literature. Most lipomas are asymptomatic and discovered during an autopsy but may cause chest pain, dyspnea, palpitations, syncope, arrhythmias, and even sudden death.^2^^,^^3^ Our patient’s symptoms are probably related to decreased cardiac output caused by the tumor impeding right atrial/ventricular filling. Most lipomas are detected during an echocardiographic study. MRI with fat suppression is very helpful to characterize the mass along with a computed tomography scan. Positron emission tomography/computed tomography, by showing a low metabolic activity, favors a benign nature of a tumor. Surgical excision is indicated for symptomatic lipoma.

Most cases of lipomas in the literature report normal ECGs or even do not comment on the ECG at all. An isolated P pulmonale, in the absence of chronic lung or right heart disease, is almost never reported. Intriguingly, the right atrium did not appear to be enlarged on imaging studies. The depolarization of sheets of atrial myocytes interspaced between fat cells in such a large tumor may explain the large P-wave. To our knowledge, this is the first description of a right atrial lipoma causing a large P pulmonale on an ECG.

## Conclusions

Cardiac tumors are rare and frequently asymptomatic. A 2-dimensional echocardiogram remains a readily available and simple test to do. Surgical resection is indicated for symptomatic tumors. Even cytologically benign tumors are more frequent; their location and size may pose challenges at the time of surgery.

## Funding Support and Author Disclosures

The authors have reported that they have no relationships relevant to the contents of this paper to disclose.

## Appendix

Video 1 An Echocardiogram View of Right Ventricular Inflow Showing a Large, Mobile, Rounded Mass in the Right Atrium The mass has no stalk. It is not attached to the interatrial septum, nor to the tricuspid valve.

Video 2 An Echocardiogram 4-Chamber View Showing the Same Large, Mobile, Rounded Mass in the Right Atrium

## References

[1] D’Souza J., Shah R., Abbass A., Burt J.R., Goud A., Dahagam C. (2017). Invasive cardiac lipoma: a case report and review of literature. BMC Cardiovasc Disord.

[2] Shu S., Wang J., Zheng C. (2021). From pathogenesis to treatment, a systemic review of cardiac lipoma. J Cardiothorac Surg.

[3] Genovese D., Gasparetto N. (2021). Proarrhythmic side of cardiac lipoma. Circ Cardiovasc Imaging.

